# Distribution of Y-Receptors in Murine Lingual Epithelia

**DOI:** 10.1371/journal.pone.0046358

**Published:** 2012-09-26

**Authors:** Maria D. Hurtado, Andres Acosta, Paola P. Riveros, Bruce J. Baum, Kirill Ukhanov, Alicia R. Brown, Cedrick D. Dotson, Herbert Herzog, Sergei Zolotukhin

**Affiliations:** 1 Department of Pediatrics, University of Florida, Gainesville, Florida, United States of America; 2 Department of Medicine, University of Florida, Gainesville, Florida, United States of America; 3 Molecular Physiology and Therapeutics Branch, National Institute of Dental and Craniofacial Research (NIDCR), National Institutes of Health (NIH), Bethesda, Maryland, United States of America; 4 Departments of Neuroscience and Psychiatry, Center for Smell and Taste, University of Florida, Gainesville, Florida, United States of America; 5 Neuroscience Program, Garvan Institute of Medical Research, Sydney, Australia; German Institute for Human Nutrition, Germany

## Abstract

Peptide hormones and their cognate receptors belonging to neuropeptide Y (NPY) family mediate diverse biological functions in a number of tissues. Recently, we discovered the presence of the gut satiation peptide YY (PYY) in saliva of mice and humans and defined its role in the regulation of food intake and body weight maintenance. Here we report the systematic analysis of expression patterns of all NPY receptors (Rs), Y1R, Y2R, Y4R, and Y5R in lingual epithelia in mice. Using four independent assays, immunohistochemistry, *in situ* hybridization, immunocytochemistry and RT PCR, we show that the morphologically different layers of the keratinized stratified epithelium of the dorsal layer of the tongue express Y receptors in a very distinctive yet overlapping pattern. In particular, the monolayer of basal progenitor cells expresses both Y1 and Y2 receptors. Y1Rs are present in the parabasal prickle cell layer and the granular layer, while differentiated keratinocytes display abundant Y5Rs. Y4Rs are expressed substantially in the neuronal fibers innervating the lamina propria and mechanoreceptors. Basal epithelial cells positive for Y2Rs respond robustly to PYY_3–36_ by increasing intracellular Ca^2+^ suggesting their possible functional interaction with salivary PYY. In taste buds of the circumvallate papillae, some taste receptor cells (TRCs) express YRs localized primarily at the apical domain, indicative of their potential role in taste perception. Some of the YR-positive TRCs are co-localized with neuronal cell adhesion molecule (NCAM), suggesting that these TRCs may have synaptic contacts with nerve terminals. In summary, we show that all YRs are abundantly expressed in multiple lingual cell types, including epithelial progenitors, keratinocytes, neuronal dendrites and TRCs. These results suggest that these receptors may be involved in the mediation of a wide variety of functions, including proliferation, differentiation, motility, taste perception and satiation.

## Introduction

Neuropeptide Y (NPY), Peptide YY (PYY), and Pancreatic Polypeptide (PP) belong to a family of peptides sharing similar hairpin-like PP-fold structural homology and evolutionary history [1]. NPY is widely expressed in the central as well as in the peripheral nervous system; PYY is released mostly by L-endocrine cells in the distal gut epithelia, while PP is produced by specialized cell in the pancreas. These peptides mediate various complementary and often opposing metabolic functions such as appetite and satiation, energy intake and expenditure; cell proliferation, migration, and differentiation; neuromodulation, angiogenesis, osteogenesis, and many other biological processes. This diversity of functions is mediated through the extensive redundancy of PP-fold peptides binding to five known receptors (Rs), Npy1r, Npy2r, Npy4r, Npy5r, and Npy6r (hereafter referred to as Y1R, Y2R, Y4R, Y5R, and y6R). The YRs belong to the rhodopsin-like superfamily of metabotropic G Protein-Coupled Receptors (GPCRs). All YRs act through G_i/o_ signaling pathway inhibiting cAMP synthesis, activating Protein Kinase C (PKC), Mitogen-Activated Protein Kinase (MAPK), or Phospholipase C (PLC), thus inducing release of intracellular Ca^2+^. In addition, YR downstream signaling modulates the conductance of membrane Ca^2+^ and inwardly rectifying K^+^ (GIRK) channels. The pharmacological redundancy of NPY family receptors is further increased by the action of dipeptidyl-peptidase-IV (DPPIV), a serine exopeptidase that truncates NPY and PYY at their N termini producing peptides NPY_3–36_ and PYY_3–36_ and thereby changing their binding specificity.

Adding more complexity to the physiological role of PP-fold peptides, we have recently documented that PYY_3–36_ is present in saliva and showed the expression of its preferred receptor, Y2R, in the basal layer of the progenitor cells of the tongue epithelia and von Ebner's gland [2]. Although the innate physiological functions of salivary PYY_3–36_ are yet to be fully determined, we have presented data that support a role of salivary PYY in the modulation of food intake (FI) and in the accumulation of body weight. This anorexigenic effect is apparently mediated through the activation of Y2 receptors in a subpopulation of cells in the oral mucosa [2]. Other groups have shown the presence of NPY in human saliva [3] and the expression of the NPY gene in the taste receptor cells (TRCs) in the rodent [4]. Given the widespread pattern of expression of PP-fold peptides and cognate YRs in other tissues, and taking into account their pleiotropic functions and the redundancy of their interactions, it was important to determine whether other members of the NPY gene family are also expressed in the oral cavity. The purpose of the current investigation, therefore, was to identify the expression of genes coding for most studied members of the YR family (Y1R, Y2R, Y4R, Y5R) in tongue epithelia cells.

## Materials and Methods

### *In vitro* YR antibody validation

HEK 293 cells were transfected with plasmids expressing murine Y1R, Y2R, Y4R, Y5R, or GFP cDNAs under the control of the strong constitutive Cytomegalovirus-Chicken b-actin (CBA) promoter. Two days after transfection, cells were fixed on cover slips and subjected to immunocytochemistry (ICC) analysis using the respective antibodies and conditions employed for YR detection in tissue samples (see Immunostaining section, below). The source of all antibodies, dilutions, and controls is listed in Table 1.

**Table 1:** Antibodies used for immunolocalization studies.

Antibody	Immunogen	Host	Supplier	Dilution	Specificity/Control
Anti-Y1R	Synthetic peptide sequence corresponding to amino acids 356–382 of the rat NPY Y1R, aa sequence TDVSKTSLKQASPVAFKKISMNDNEKV.	Rabbit	Immunostar (Hudson Wiconsin, USA; cat No. 24506)	1∶100 (using TSA Kit)	Staining absent when primary or secondary antibodies omitted. The antibody was characterized by immunohistochemistry and Western blot. Western blot showed one immunoreactive band of 40 kD and a single high molecular weight band, presumably a precursor molecule (manufacturer statement). Preincubation of the antibody with an excess of the synthetic peptide blocked staining. Immunohistochemical staining of rat brain correlates well with Northern analysis, in situ hybridization and receptor autoradiography. BlastP database sequence homology searches confirmed that this sequence is unique to rat, mouse and human NPY Y1 receptors.
Anti-Y2R	Synthetic peptide from the cloned NPY Y2R, aa sequence CTDSFSEATNV-COOH	Rabbit	Neuromics (Edina, MN, USA; cat. No. RA14112)	1∶3000 (using TSA Kit)	Staining absent when primary or secondary antibodies omitted, or in NPY Y2 receptor KO. Use of this antibody has been reported previously. Western blot analysis on hippocampal membrane fractions revealed a single band of 44 kDa (Stanic et al, J Comp Neurol 2011, v.519, p. 1219–1257).
Anti-Y4R	Amino acids 74–116 near the N-terminus of human NPY4R (Protein accession # P50391)	Rabbit	Santa Cruz Biotechnology, Inc. (cat. No. sc-98934)	1∶1600 (using TSA Kit)	Staining absent when primary or secondary antibodies omitted.
Anti-Y5R	Synthetic peptide conjugated to KLH derived from within residues 150–250 of Human NPY5R. The exact immunogen sequence is known to the authors, but is withheld from the manuscript following manufacturer's request.	Rabbit	Abcam; (cat. No ab43824)	1∶800 (using TSA Kit)	This antibody gave a positive signal in the following Lysates: Mouse Fetus (14 day old) Tissue, Mouse Spinal Cord Tissue, THP1 Whole Cell, Caco 2 Whole Cell, Rat Ovary Tissue, Rat Colon Tissue, and Mouse Skeletal Muscle Tissue (manufacturer statement). Medeiros et al, Int J Cancer, 2012, 131(2):276-86
Anti-NCAM		Rabbit	Millipore (Temecula, CA, USA; cat. No. AB5032)	1∶500	Staining absent when primary or secondary antibodies omitted. Use of this antibody has been reported previously. Acosta et al, PLoS ONE, 2011, 6(10):e26137.

### Mice

#### Ethics Statement

This study was approved by the Institutional Animal Care and Use Committee (IACUC) at the University of Florida (UF, Permit Number: 03059 “Modulation of taste sensitivity by PYY signaling”). All procedures were done in accordance with the principles of the National Research Council's guide for the Care and Use of Laboratory Animals. All surgery was performed under sodium pentobarbital anesthesia, and all efforts were made to minimize suffering. Tissues were collected from sacrificed animals and immediately frozen in O.C.T. Compound. Mice were housed at 22–24°C in a 12 hours dark/light cycle and had access to water and food *ad libitum* unless indicated otherwise.

### Tissues

Tongues and brains were harvested from wild type C57Bl/6 male mice from Charles River, as well as from homozygous Y1R KO [5] and Y2R KO [6] mice. Both KO strains are maintained at the UF animal facility. Genotype was confirmed using the following genotyping primer sets: Y1R KO: primer mY1-H 5′-TGGCAAAACAGGTCCCTG-3′; and primer mY1-P5 5′- CTAGCCAGTTGGTAATGG-3′; Y2R KO: primer NHE-3A 5′-TTAACATCAGCTGGCCTAGC-3′, and primer NHE-6 5′-GGAAGTCACCAACTAGAATGG-3′.

### Immunostaining

#### YR immunoreactivity staining

For specific information on the source of all antibodies, dilutions, and controls please see Table 1. Tissues were harvested from overnight fasted animals and immediately frozen in O.C.T. Compound. Four µm thick coronal sections were cut using a cryostat (Leica CM3050 S; Leica Microsystems, Nussloch GmbH, Germany), mounted onto Fisher Superfrost Plus slides and post-fixed in 4% paraformaldehyde for ten minutes. YR immunolocalization was conducted utilizing the tyramide signaling amplification (TSA) kit (Perkin Elmer). Tissues were blocked in 0.3% H_2_O_2_ in Tris-buffered saline (TBS) for 30 minutes at room temperature (RT) to eliminate endogenous peroxidase activity, followed by blocking with 0.1 M Tris-HCl, pH 7.5, 0.15 M NaCl and 0.5% Blocking Reagent from Perkin Elmer; for 60 minutes at room temperature to reduce nonspecific antibody binding. Sections were then incubated with primary rabbit anti-YR in TNT (0.1 M Tris HCl, pH 7.5, 0.15 M NaCl, 0.05% Tween 20) overnight at 4°C, followed by secondary goat anti-rabbit IgG (Fab')2 conjugated to horseradish peroxidase (Abcam; 1∶1000 for 60 min at RT). Staining was detected using Fluorescein provided in the TSA kit (1∶300 for 7 min at RT). Negative controls were run concomitantly. All sections were counterstained with DAPI (4′,6-diaminidino-2-phenylindole).

#### Mirror section staining

To overcome the technical limitations imposed by the origins of antibodies (all four a-YRs were raised in rabbits), we utilized a mirror section staining method. In brief, the first section is mounted with its inner surface turned upwards on one slide, whereas the subsequent adjacent section is mounted on the next slide with its outer surface upwards. In this way, the complementary faces could be hybridized to two different antibodies without concern about the secondary antibody cross-reactivity. Although not entirely identical, the characteristic structures of the epithelial layers on the two separate slides provide sufficient visual guidance to identify distinctive cell layers or even particular cells.

#### YRs/NCAM (neural cell adhesion molecule) double staining

Because YR and NCAM primary antibodies were raised in rabbits, double immune-labeling was performed using a modified indirect immunofluorescence (IF) protocol and the TSA kit allowing for the localization of two antigens in the same tissue specimen when both primary antibodies produced in the same host [4], [7]. The use of TSA permits control for type I and II interference [4]. A first primary antibody is used at very low concentrations that can only be detected with amplification kits such as TSA and not with a conventional protocol (interference I). A first secondary antibody is then used to detect this first primary, however only the Fab' fragment can be used in order to avoid binding of a second primary antibody to the first secondary antibody (interference II). This protocol has been fully described by Kaya et al. [7] and has been followed without modifications. Specifically, immediately after detection with the TSA Fluorescein, sections were extensively washed in TNT and then blocked with 10% donkey serum in TNT for 60 min at RT. Tissues were subsequently incubated with the second primary antibody rabbit anti-NCAM (Millipore; 1∶250 in 10% donkey serum overnight at 4°C), and visualized, using standard methods, with AF555 donkey anti-rabbit IgG (Invitrogen, 1∶1000 in TNT for 60 min at RT). All double-labeled sections were counterstained with DAPI.

### *In situ* hybridization

*In situ* hybridization (ISH) was performed using QuantiGene® ViewRNA ISH Tissue Assay (Affymetrix) following the manufacturer's protocol. Gene-specific probe sets for murine Y1R, Y2R, Y4R, and Y5R mRNAs consisting, on average, of 20 different probe pairs were custom-designed and synthesized by Affymetrix. Where available, the respective tissues from the germline KO mice (Y1R, Y2R) were used as negative control samples. For Y4R and Y5R hybridizations, a no-probe sample was utilized as a negative control per the Affymetrix manual's recommendations. Hybridized target mRNAs were visualized using confocal fluorescent microscopy, or by bright field microscopy.

### Ca^2+^ imaging

Adult C57Bl/6 mice were used in this study. Following sacrifice, the tongue was quickly dissected in the ice-cold modified artificial cerebrospinal fluid (ACSF) saturated with 95% O_2_ and 5% CO_2_ that contained: 120 mM NaCl, 25 mM NaHCO_3_, 5 mM KCl, 1.25 mM Na_2_HPO_4_, 1 mM MgSO_4_, 1 mM CaCl_2_, 10 mM glucose, 305 mOsm. Small tissue blocks were embedded in 4% low-melting agarose and sectioned using Leica V1000 vibratome. Lingual slices were placed in a perfusion chamber (RC-26, Warner Instruments). Slices were incubated with 10 µM fura-2/AM for 30 min at 37°C. The chamber was then mounted on the stage of the upright microscope (Zeiss Axioskop FS2) equipped with a water-immersion 40× objective lens (Neofluar, NA1.4). Solutions were applied locally using a fine bore capillary attached to a 8-channel Teflon manifold (BioLogic, France) under the control of the perfusion system (VC-6, Warner Instruments) controlled by pClamp 9.2 (Molecular Devices). Images were acquired with a cooled CCD camera (ORCA R2, Hamamatsu) under the control of Imaging Workbench 6 software (INDEC Systems). The illumination system (Lambda DG-4, Sutter Instruments) was coupled to the microscope with a liquid light guide. Ratiometric imaging was performed using a Fura-2 filter set (excitation at 340 nm or 380 nm, emission at 510 nm, dichroic mirror 435 nm). Before calculating the ratio at 340/380 nm all images were corrected for background fluorescence. Off-line analysis and image processing was performed using Imaging Workbench 6, ImageJ 1.46 (available at http://rsbweb.nih.gov/ij/index.html) and ClampFit 9.2 (Molecular Devices).

### RNA extraction and RT-PCR analysis

Approximately 1×2 mm section of the non-gustatory tongue epithelium anterior to the circumvallate papillae (CV) was cut with microscissors under a dissecting microscope; the muscle layer was removed. A 1 mm punch (biopsy punch- Miltex, Inc, York, PA) was used to collect the CV. Although the majority of the proximal epithelia tissues and the ventral muscle were removed, the samples likely included some non-gustatory tissue. Total RNA was extracted with Trizol (Invitrogen, Carlsbad, CA); DNA was digested with RNase free DNase (Qiagen Inc, Valencia, CA) followed by RNA cleanup with the RNeasy Micro kit (Qiagen). One microgram of RNA was reverse-transcribed with Superscript III (Invitrogen). Products were amplified for 35 cycles with gene-specific primers (Table 2) utilizing RedTaq Ready Mix (Sigma-Aldrich, Saint Louis, MO). “No cDNA” samples were prepared for each primer set. DNA contamination was tested with control, intron-spanning primers. Products were resolved in a 2% agarose gel.

**Table 2:** NPY family RT-PCR primers.

Accession#	Name	Forward	Reverse	Amplicon (bp)	Tm	Span Intron
NM_010934.4	Y1R	TGGCTTTTGAAAATGATGACTG	ATAAGCGAGAGCCAAGGTGA	65	60	No
NM_008731.3	Y2R	TTGGCAACTCCCTGGTAATC	TTTCCACTCTCCCATCAAGG	155	60	No
NM_008919.4	Y4R	GGGCCCAGATAGGTTGGCAAGAGA	CCCTTGCAGCTCAAGCCACAAAGT	128	65	No
NM_016708.3	Y5R	AAGACGGCATGCGTGTTAC	TGGAACGGTTAGGTGCTTCT	63	65	No
NM_145435.1	PYY	GGCACTTCATATCTCGGTGTCTCGG	TGAACACACACAGCCCTCCAGTCT	55	62	No
NM_023456.2	NPY	TCATCTCATCCCCTGAAACC	CGGAGTCCAGCCTAGTGGT	66	61	No

## Results

Previously, we have demonstrated the expression of Y2Rs in basal lingual epithelial cells [2]. The dorsal surface of the tongue is covered by a specialized mucosa consisting of keratinized stratified epithelium [8]. Stratified epithelium is characterized by the high turnover rate of cells in response to mechanical and chemical contacts. Because of the known functions of YRs in cell proliferation [9], it was of interest to explore whether epithelial cells express these receptors.

### RT-PCR analysis

**Figure 1:**
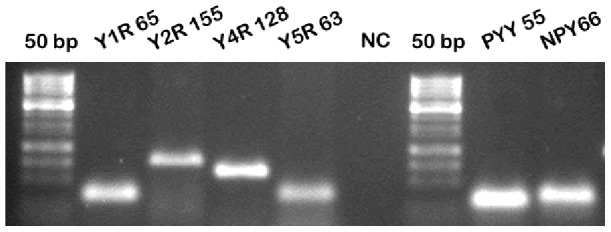
RT-PCR analysis of the expression of NPY family members in the lingual epithelia. The names of the genes and the expected PCR fragments sizes (in bp) are shown above the respective lanes. NC – negative (no cDNA) control.

To characterize the expression of other members of YR family, we performed RT-PCR analysis using RNA isolated from the non-gustatory lingual epithelium, as well from the taste buds. All members of the NPY family, including four cognate receptors, Y1R, Y2R, Y4R, and Y5R, as well as their respective agonists NPY and PYY were expressed in taste buds (Fig. 1) and in non-gustatory epithelium (data not shown). The identities of PCR fragments were confirmed by sequence analysis (data not shown). In addition several pairs of primers were utilized for each member of the NPY family, including those designed to amplify intron-spanning fragment of the respective gene. PCR amplification was mRNA-specific and no chromosomal DNA contamination was detected (data not shown).

### ISH analysis

**Figure 2:**
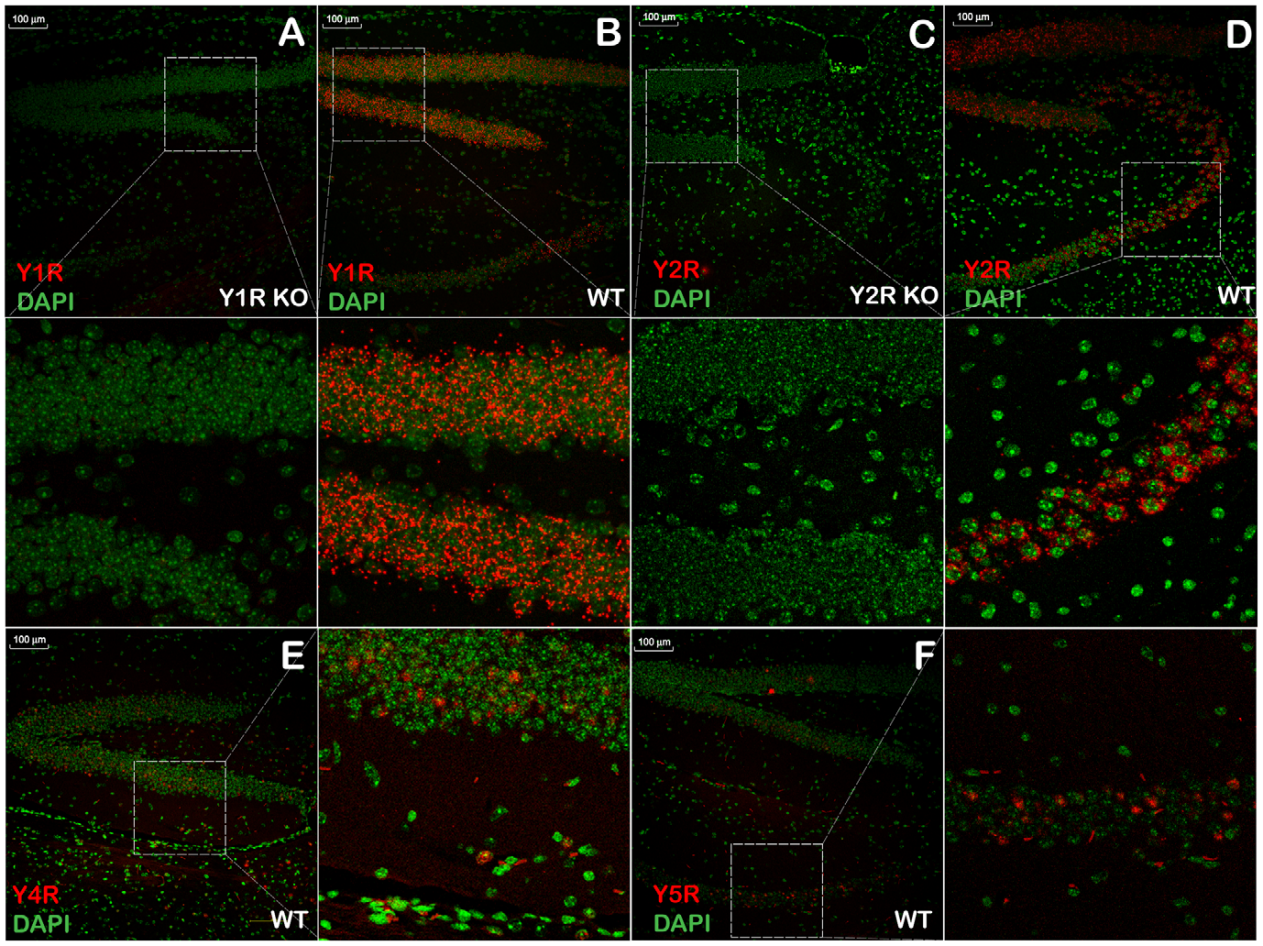
Validation of *in situ* hybridization (ISH) probe sets by ISH analysis of Y-receptor (YR) gene expression in the dentate gyrus of the mouse brain. **A** and **C** – negative control samples, Y1R KO and Y2R KO, respectively; **B**, **D**, **E**, and **F** – positive control samples, WT C57Bl/6 mice. Representative fields outlined by the dashed rectangles in panels **A** through **F** are shown as close-up images either below the respective panels (panels **A**, **B**, **C**, and **D**), or to the right of the panel (panels **E** and **F**). Red dots show fluorescently visualized YR mRNAs. DAPI-stained nuclear DNA is shown in pseudo-colored green hue for better viewing.

To validate the RT-PCR results using an independent method, we analyzed the expression of the Y1R, Y2R, Y4R, and Y5R genes using an ISH assay in coronal sections of lingual tissues. Each custom-designed probe set included, on average, 20 oligonucleotides pairs complementary to the target mRNA in the regions divergent from other family members. As such, each probe set represented a highly specific reagent hybridizing to the respective target at high selectivity. The protocol was validated in brain sections, while tissues from KO mice, when available, were used as negative controls (Fig. 2A, C). Alternatively, no-probe control tissues from C57Bl/6 mice were utilized. All control tissues exhibited no non-specific binding (data not shown). The positive control hybridization with the brain tissues resulted in a pattern consistent with the data described in several publications [10], [11], [12], [13], [14], [15]. These latter results clearly demonstrated the specificity of the YR probe sets.

**Figure 3:**
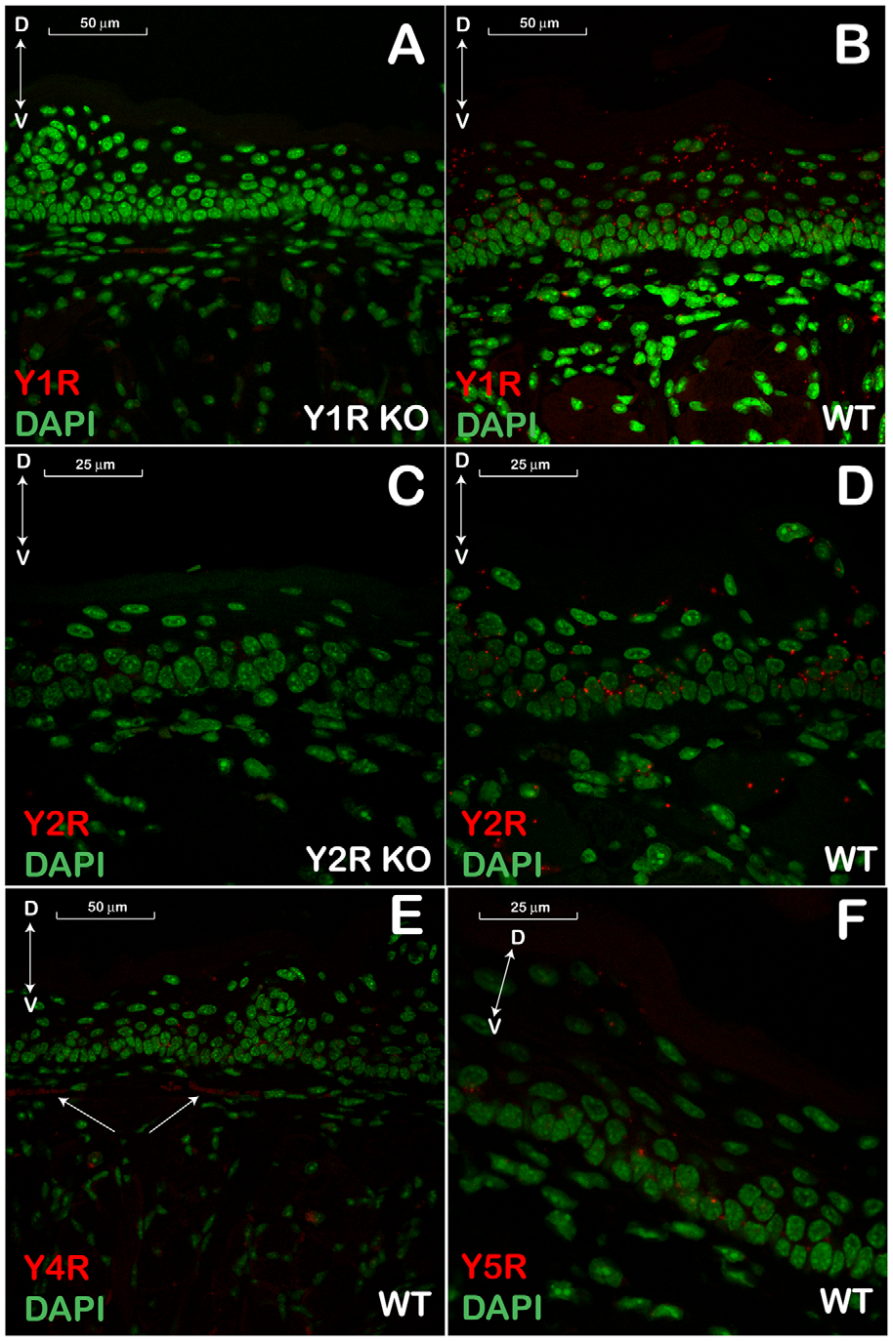
*In situ* hybridization analysis of Y-receptor (YR) gene expression in the dorsal lingual epithelium. **A** and **C** - (−) control samples, Y1R KO and Y2R KO, respectively; **B, D, E, and F** – wt C57Bl/6 samples. Bidirectional arrows indicate dorsal/ventral (D/V) coordinates of the section. One-direction arrows in panel E point at Y4R-positive neuronal fiber/s. Red dots show fluorescently visualized YR mRNAs. DAPI-stained nuclear DNA is shown in pseudo-colored green hue for better viewing.

In the dorsal epithelial layer of the tongue, the pattern for YR mRNAs expression, appeared to be different for different subtypes: Y1R expression was more prevalent in the superficial layers, including keratinocytes (Fig. 3B), while Y2R was more pronounced in, but not restricted to, the basal epithelial layer (Fig. 3D). Interestingly, we detected neuronal fibers positive for Y4R-encoding mRNA in the subepithelial region close to the basal laminae (Fig. 3E, arrows). Y5R mRNA staining was faint but clearly detectable in all layers of the epithelia, including keratinocytes (Fig. 3F). Similar to the brain tissue negative controls, the nonspecific staining in the negative control KO sections was negligible (Fig. 3A, C), as well as in the no-probe control sections with tissue from WT mice (data not shown).

**Figure 4:**
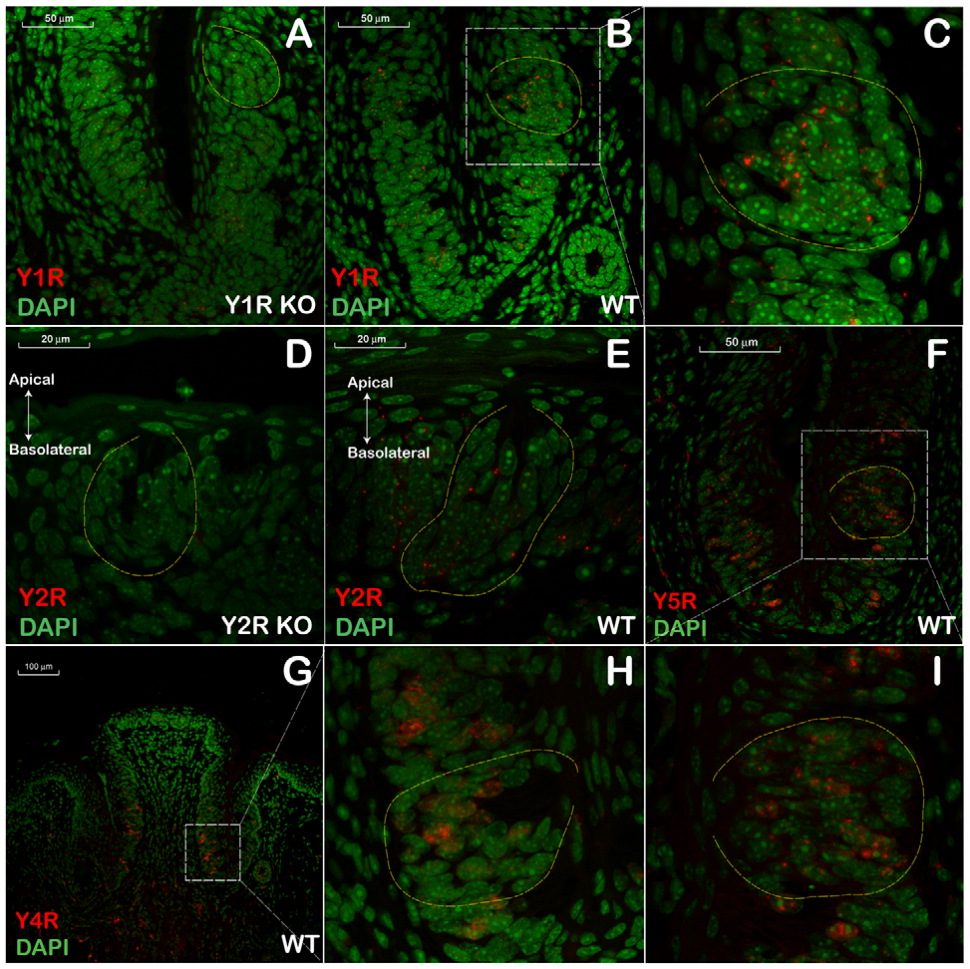
*In situ* hybridization analysis of Y-receptor (YR) gene expression in the circumvallate papillae. **A** and **D** – negative control samples, Y1R KO and Y2R KO, respectively; **B**, **E**, **F**, and **G** - samples from wt C57Bl/6 hybridized to Y1R, Y2R, Y5R, and Y4R probe sets, respectively; **C**, **H**, and **I** – close up images of the representative areas from the respective panels (**B**, **G** and **F**, respectively) outlined by white dashed rectangles. The borders of several taste buds are outlined with yellow dashed lines. Red dots show fluorescently visualized Y-receptor mRNAs. DAPI-stained nuclear DNA is shown in pseudo-colored green hue for better viewing.

In the gustatory epithelium from the CV, the expression of the respective Y1R, Y2R, Y4R, and Y5R mRNAs also showed an absence of non-specific binding from both KO control mice (Fig. 4A, D) and no-probe control sections from WT mice (data not shown). The distribution of YR expression was not restricted to any particular cell population within the taste bud, but rather appeared in many cells localized at the apical and basolateral portions of the CV (Fig. 4B, C E, F, G, H, I).

### Validation of YR antibodies

**Figure 5:**
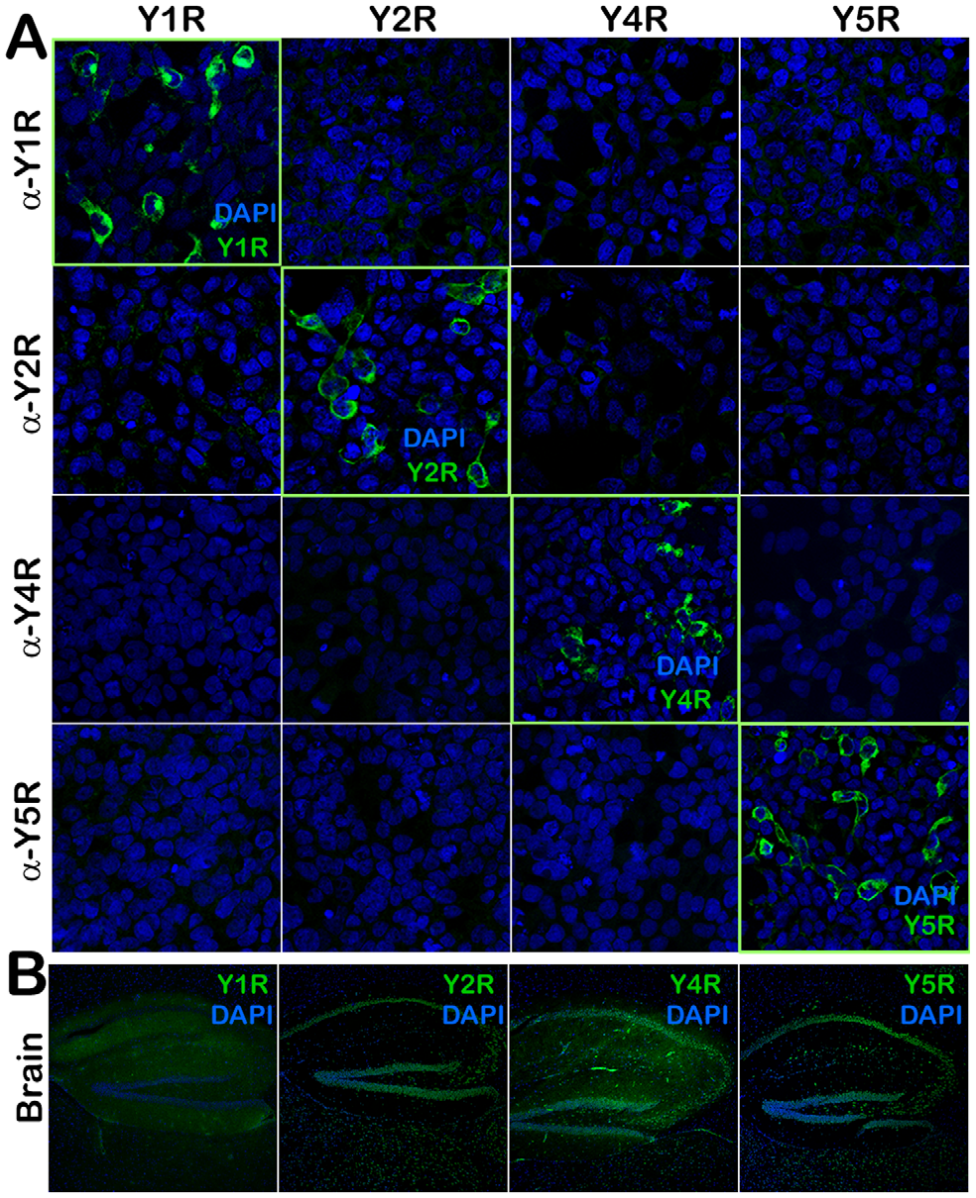
Validation of Y-receptor (YR) antibodies. **A** - Immunofluorescence (IF) analysis of 293 HEK cells expressing murine YR cDNAs. Columns – cells transfected with Y1R, Y2R, Y4R, Y5R-expressing plasmids, respectively. Rows – cells on cover slips incubated to a-Y1R, a-Y2R, a-Y4R, or a-Y5R antibodies, respectively. Please note peripheral (membrane-associated) localization of YRs. **B** - IF analysis of YRs in the dentate gyrus (coronal sectioned planes) of mouse brain. The diffuse staining for Y1R reflects the distribution of Y1R positive neuronal fibers rather than cell bodies.

To characterize the expression of YR subtypes in a cell-specific manner, we next conducted an IHC analysis. YRs belong to GPCR family of receptors and, as such, are homologous to each other as well as to other GPCRs [16]. It is recognized that a significant fraction of commercially available GPCR antibodies lack specificity and selectivity resulting in binding to other subtypes within the family [17], [18]. It was therefore essential to validate the antibodies used herein prior to conducting an IHC experiment. Utilizing ICC analysis in HEK 293 cells expressing Y-receptors subtypes with the conditions employed for YR detection in tissue samples, we clearly demonstrated that each antibody reagent interacts exclusively with its respective antigen, i.e. Y1R, Y2R, Y4R, or Y5R with no detectable cross hybridization to any other YR subtypes (Fig. 5A).

We selected the hippocampal dentate gyrus region as a positive control tissue because of its robust expression of YRs in a very specific and well-characterized fashion. Staining of brain sections revealed patterns of YR-positive neuronal cell bodies and fibers that are comparable to those previously reported (Fig. 5B) [10], [11], [13], [14], [15]. In particular, expression patterns of Y1R and Y2R appeared to be complementary, i.e. high levels of Y1R expression in one region corresponded to the low levels of Y2R, and vice versa [11].

### Immunohistochemical (IHC) analysis

**Figure 6:**
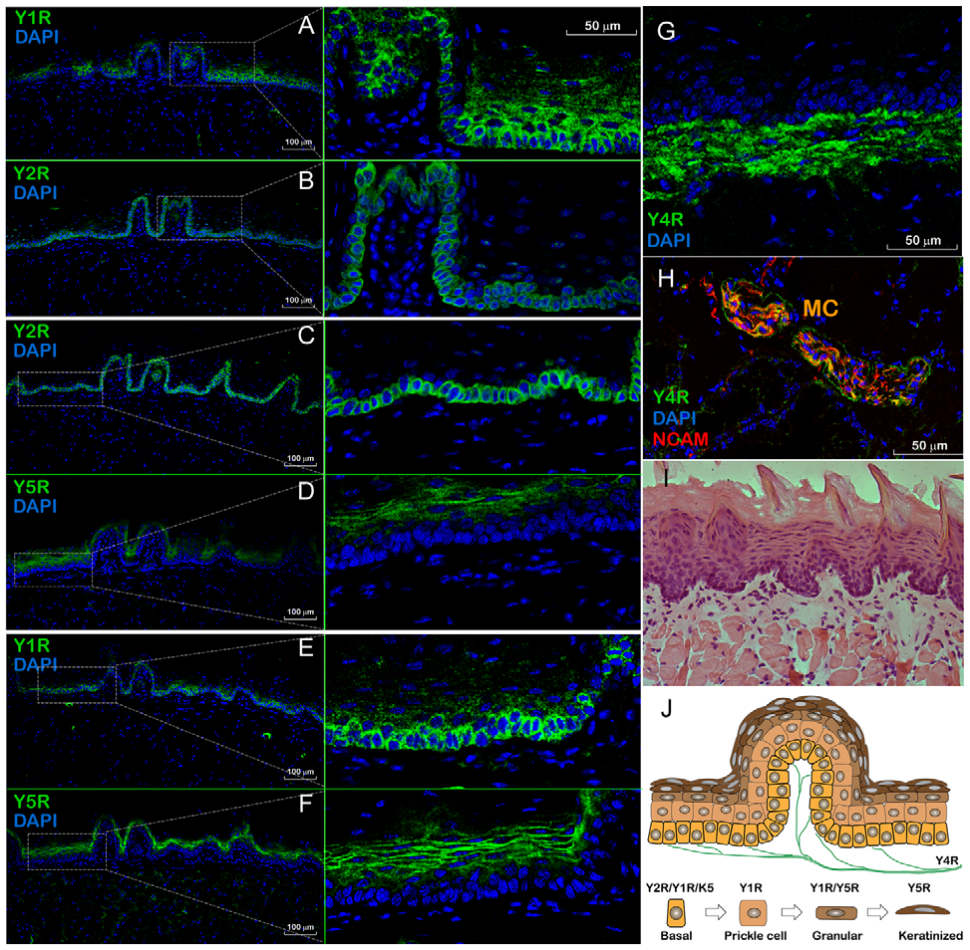
Immunolocalization of Y1, Y2, Y4, and Y5 receptors (Rs) in the dorsal epithelium of murine tongue. Mirror section pairs (Panels **A** and **B**, **C** and **D**, **E** and **F**) were hybridized to the respective YR antibody (green), followed by DAPI counterstain (blue), as indicated in the upper left corner of each panel. For better viewing, the confocal images in **B**, **D**, and **F** were reflected horizontally. Representative areas of the epithelium, positive for either YR (dashed rectangles in the left-sided panels), are shown as close-up images on the right next to each respective panel. The irregular columned structures at the epithelial surface are transversely sectioned filiform papillae. **G -** Y4R-positive neuronal fibers (green) are located in the subepithelial region underlying the basal laminae. **H** – co-localization of Y4R and NCAM (red) immunoreactivity within mechanoreceptors of Meissner corpuscles (MC). As a morpho-histological reference of the dorsal lingual epithelium structure, an hematoxylin and eosin stained section is shown in panel **I**. Panel **J** shows a hypothetical diagram of a lingual dorsal epithelium layer and the differentiation/migration lineage of cell types expressing respective YR subtypes. K5 – cytokeratin-5 [2].

Using a mirror section staining protocol, we next determined that morphologically different layers of lingual epithelia expressed YRs in a distinctive yet overlapping pattern. Three separate complementary pairs of mirror sections were analyzed by hybridization to YR antibodies: Y1R and Y2R (Fig. 6A and B, respectively); Y2R and Y5R (Fig. 6C and D, respectively); Y1R and Y5R (Fig. 6E and F, respectively). The basal epithelial cell monolayer expressed both Y1Rs (Fig. 6A, E) and Y2Rs (Fig. 6B, C). Y2R expression appeared to be restricted only to this monolayer (Fig. 6C, right panel), while Y1R, on the other hand, was present in the parabasal prickle cell layer, and the granular layer (Fig. 6A, right panel). Differentiated keratinocytes displayed very low levels of Y1R protein, however, they displayed considerable expression of Y5Rs (Fig. 6F, right panel).

Unlike Y1R, Y2R, or Y5R, the expression of Y4R was not detected in the basal, or keratinized epithelial cells. Instead, it was restricted to the neuronal fibers extending within the subepithelial region close to the basal laminae of the lingual epithelia (Fig. 6G). Very few YR4-positive fibers were also positive for NCAM (data not shown) suggesting that the majority of these projections represented intraepithelial axons with free nerve endings. In addition, Y4R-positive neuronal fibers were also abundant in some areas of the lamina propria, in particular, in the fibers innervating mechanoreceptors (Meissner corpuscles) that were also positive for NCAM (Fig. 6H).

### Functional assay for YR-positive epithelial cells

**Figure 7:**
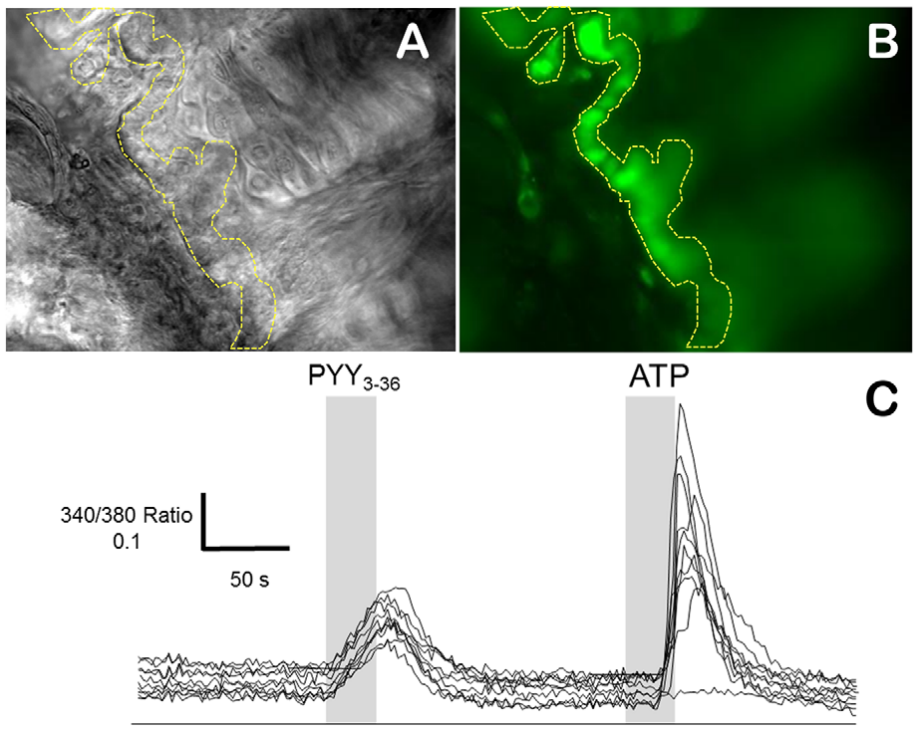
PYY_3–36_ induces intracellular Ca^2+^ in the lingual basal cell epithelium layer. **A** – differential interference contrast micrograph of the agarose embedded acutely-obtained mouse lingual slice. The basal layer of epithelial Y1R/Y2R/K5-positive cells is outlined by a yellow dashed line. **B** – The same slice was loaded with a Ca^2+^ sensitive indicator dye, fura-2/AM. A static fluorescence image was captured at 380 nm excitation showing strong fura-2 loading in the basal cellular layer. **C** – Representative traces recorded from 13 individual cells selected in the slice shown in **B**. PYY_3–36_ (40 µg/ml) was applied by a local microperfusion for 30 sec indicated with the shaded box. ATP (20 µM) was used as a positive functional control. The data were collected from 38 representative cells individually analyzed in 3 slice preparations from two different animals.

Previously, it has been shown that YRs mediate their responses through the G_i/o_ signaling pathway, reviewed in [1]. Several second messengers produced downstream of activated Y1, Y2, and Y4Rs include also inositol triphosphate, which is responsible for the increase in intracellular Ca^2+^ concentration [19], [20], [21]. To assess whether YR-positive cells in the dorsal epithelial layer could respond functionally to salivary PYY_3–36_, we conducted *ex vivo* Ca^2+^-imaging experiments on lingual sections loaded with fura-2/AM, a ratiometric fluorescent dye that binds to free intracellular Ca^2+^. Indeed, incubation of sections with PYY_3–36_ (40 µg/ml; applied locally) induced a clear elevation in intracellular Ca^2+^ concentration in the basal cellular monolayer (Fig. 7A, B). The PYY_3–36_ activation was rather uniform and led to a slow intracellular Ca^2+^ elevation in a majority of cells in the basal cellular layer (Fig. 6C). Qualitatively, a very similar pattern of response was observed in different slices loaded with a non-ratiometric Ca^2+^ indicator fluo-3/AM (data not shown). As a positive control to account for the functional integrity of cells we used ATP (20 µM) to activate strong Ca^2+^ signaling through its binding to purinergic receptors that are ubiquitously expressed in most cellular types (Fig. 7C) [22]. Application of ATP evoked Ca^2+^ signaling with a significant delay and variable in amplitude compared to the PYY-induced signal, i.e., shorter delay and more uniform in shape (Fig. 7C).

### Expression of YRs in taste bud cells

**Figure 8:**
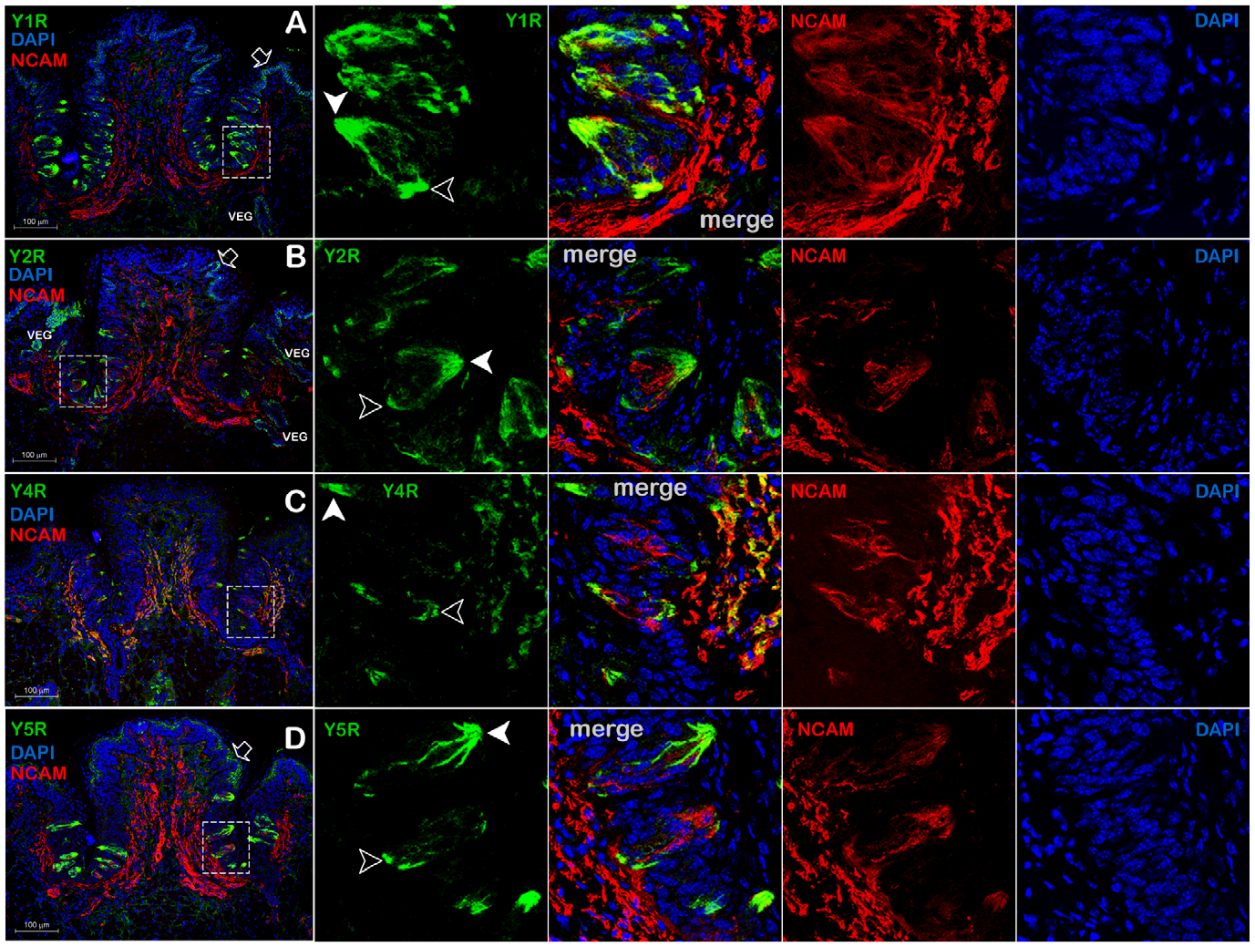
Immunolocalization of Y receptors (YRs) in taste receptor cells (TRCs). Panels **A**, **B, C, and D** demonstrate Y1R-, Y2R-, Y4R-, and Y5R-positive TRCs, respectively. Areas within the dashed rectangle are shown as a series of magnified images to the right, stained for YRs (green), NCAM (red), DAPI (blue), respectively, with the merged images shown between YRs and NCAM. Open block arrows in the panels **A**, **B**, and **D** point at the YR-positive epithelial cells; filled arrowheads show apical parts of the YR-positive TRCs; open arrowheads – basolateral parts; VEG – von Ebner's gland. The majority of NCAM-positive intragemmal fibers are also Y4R-immunopositive (Panel **8C**), but do not appear to express Y1Rs, Y2Rs, or Y5Rs.

To characterize whether TRCs in the CV expressed YRs, we used an IHC assay in tongue sections harvested from mice sacrificed after a 24-hour fast. In agreement with data gathered from the dorsal lingual epithelial layer (Fig. 6A, B), epithelial cells forming the CV's outer edges also expressed Y1R, Y2R, and Y5Rs in a selective manner (Fig. 8A, B, and D, block arrows). In addition, cell monolayers lining the ducts of von Ebner's glands were also positive for Y1R and Y2Rs (Fig. 8A, B, indicated by VEG).

A subpopulation of TRCs was positive for Y1Rs (Fig. 8A), Y2Rs (Fig. 8B), Y4Rs (Fig. 8C), or Y5Rs (Fig. 8D). In contrast to the epithelial cells, the staining pattern did not delineate the entire contour of cells, showing instead preferential accumulation of YRs within the apical part of the cells (filled arrowheads in Fig. 8A–C, respective magnified images in the panels on the right). To begin to understand the putative functions of YRs, we co-stained YRs with a molecular marker, NCAM that is expressed in intragemmal nerve fibers. It appears that many Y1R, Y2R, Y4R, and Y5R-positive cells also express NCAM (Panels A through C, magnified images to the right). Only Y4R expression was detected in the taste bud intragemmal nerve fibers.

## Discussion

Recently, we showed that a member of the PP-fold family, the satiation gut peptide PYY_3–36_, is present in murine and human saliva [2]. In mice, salivary PYY_3–36_ primarily derives from plasma. It is also synthesized in the taste receptor cells in taste buds of the tongue. We have demonstrated that augmentation of salivary PYY_3–36_ induces stronger satiation and that this effect is mediated through the activation of the preferred Y2Rs expressed in the lingual epithelial cells [2]. Taking into account the presence of other PP-fold peptides in the saliva [3], the redundancy of their interactions with YRs, and the multitude of their physiological functions, it was reasonable to hypothesize that the other YRs were expressed in the oral cavity tissues. To explore the mechanisms of salivary PYY_3–36_ action and to identify peptide's molecular target substrates, we set out to characterize the expression pattern of all major members of the YR family in lingual epithelia.

The dorsal surface of the tongue is covered by a specialized mucosa consisting of keratinized stratified epithelium (for review, please see [8]). In addition to its primary function protecting the underlying tissues during mastication, it also incorporates structures with gustatory functions (CV, fungiform, and foliate papillae), mechanical structures (filiform papillae), and mechanoreceptors (Meissner corpuscles). In addition, a glandular component of the lingual epithelium includes specialized salivary glands such as von Ebner's glands. In the current report, we focus on the dorsal lingual epithelia and the CV.

Using IHC analysis and a set of validated primary antibodies for Y1Rs, Y2Rs, Y4Rs, and Y5Rs, we determined that morphologically different layers [8] of the lingual epithelia expressed YRs in a very distinctive yet overlapping pattern (Fig. 6H). The basal epithelial cell monolayer expressed both Y1Rs and Y2Rs; Y1Rs were present in the parabasal prickle cell layer and the granular layer, while differentiated keratinocytes displayed an abundance of Y5Rs. ISH analysis essentially corroborated this pattern, although the Y5R-specific probe set appeared to hybridize to the target mRNA poorly, even in control brain tissue, where Y5Rs are known to be robustly expressed [15].

The Y2R-positive basal epithelial layer, which is also cytokeratin-5 (K5)-positive [2], responded robustly to PYY_3–36_ by increasing intracellular Ca^2+^ concentration indicating the receptors' physiological activity. To identify precisely the activated Y receptor subtype, specific receptor antagonists will be needed. Meanwhile, it seems reasonable to postulate that PYY_3–36_/Y2R/G_i_ signaling in K5-positive epithelial cells plays a proliferative role inducing mitotic activity of the progenitor cells in response to the mastication-related loss of keratinized lingual epithelial cells. In this regard, the postprandial increase in salivary PYY_3–36_ [2] could provide a feedback signal inducing such proliferation. Moreover, since in oral epithelia, proliferation also occurs in parabasal cells [23], the Y1R-positive prickle cell layer could be responsive to salivary NPY as well. G_i_ signaling in K5-progenitor cells can also mediate their motility, polarity and migration towards the dorsal layer of the keratinized filiform papillae. Such YR-dependent proliferation, migration, and differentiation has been previously described for the neurons in the adult mouse brain [24], olfactory neuronal precursors [25], and human endothelial cells [26]. There is also strong evidence supporting the NPY system's angiogenic and mitogenic functions in vascular smooth muscle cells and its potential role in endothelial cells wound healing [27]. A hypothetical diagram of a lingual dorsal epithelium layer and the differentiation/migration lineage of cell types expressing respective Y receptors subtypes is shown in Fig. 6J.

Assuming that the Y1R/Y2R-positive basal epithelial cells are modulated by salivary NPY and PYY, one has to postulate a mechanism of trans-cellular transport of these hormones through the superficial layers of keratinocytes forming hard-to-penetrate tight junctions. Interestingly, one of the outcomes of the PP-fold peptides interacting with their cognate GPCR YRs is the internalization and recycling of the respective agonist/receptor complex inside the cell [28], [29], [30]. It is, therefore conceivable that salivary PP-fold agonists are transported through cell layers akin to a relay race baton where directional cell-to-cell handoff is mediated by the increased affinity towards receptors, e.g., Y5R<Y1R<Y2R. The other possible endogenous source of PP-fold peptides are oral mucosal neuroendocrine cells, also known as Merkel cells, which are distributed along the basal layer of oral keratinized epithelium (for a review, please see [31]). We also have observed multiple PYY-positive cells in the lamina propria in close vicinity to the basal epithelial layer (data not shown).

The Y4R appears to be markedly expressed in most of the neuronal fibers innervating the lamina propria and taste buds. Some of these projections, especially fibers in mechanoreceptors of Meissner corpuscles and gustatory fibers in the CV, also expressed NCAM, indicating possible synaptic connections with receptor sensory cells, while most of the subepithelial fibers were NCAM negative, indicating free nerve endings. We also detected Y4R mRNA-positive fibers using an ISH protocol (Fig. 3E), suggestive of an anterograde trafficking of the Y4R mRNA along dendrites at least in some neurons.

Gustatory papillae are distinctive structures on the dorsal tongue epithelia incorporating several types of cells including basal epithelial cells, keratinized cells, TRCs organized in tight clusters (taste buds), and gustatory neuronal fibers innervating TRCs. Epithelial cells and TRCs derive from the lingual embryonic undifferentiated epithelium and are constantly turned over in the adult animal. All tested YR subtypes were prominently expressed in murine TRCs showing preferential apical distribution within cells. This distribution would make YRs easily accessible to paracrine salivary PP-fold peptides, suggesting their possible roles in modulating taste perception [32], although some taste receptors do not display such preferential apical distribution [33]. Conversely, some YRs-positive TRCs accumulated YRs in their basolateral portion (open arrowheads in Fig. 8A–C), which makes these cells accessible to the paracrine, or endocrine PYY, or NPY, synthesized inside taste buds [2], [4].

Within each taste bud, TRCs fall into three morphological subtypes, Types I through III, which seem to correspond to functional classes (reviewed in [34]). In order to understand the functional role of YRs in TRCs, one needs to assign YR-positive TRCs to a particular cell subtype co-staining YRs with a known cell type marker. Here we show that a subpopulation of YR-positive TRCs are co-localized with NCAM, a neuronal marker expressed in some Type III cells as well as in intragemmal nerve fibers. Because of technical limitations, we were unable to quantify the exact ratio of YR/NCAM-positive TRCs due to the specific staining contours. Experiments are currently under way to characterize the precise expression pattern of YRs in Type I–III TCRs and to identify taste qualities modulated by YR subtypes.

In summary, we have conducted a systematic analysis of the expression of genes encoding members of NPY family peptides and YRs in the dorsal lingual epithelia of the mouse. We have utilized independent assays (IHC, ISH, ICC and RT-PCR) using validated primary antibodies, custom-designed target-specific probe sets, and intron-spanning PCR primers to identify cells expressing YR subtypes. Herein, we show for the first time that all members of the NPY family are abundantly expressed in specific and selective fashion in multiple lingual cell types, including epithelial progenitors, keratinocytes, neuronal dendrites, and TRCs. Because of this remarkable diversity, and because of the redundancy of agonist/receptor interactions, NPY family peptides and their cognate receptors in the oral cavity may mediate a wide variety of functions, including proliferation, differentiation, motility, taste perception, as well as satiation. All of these multiple functions and their respective molecular mechanisms are subjects of the ongoing investigations.
